# Prescriptions and Dispensing

**Published:** 1908-03-14

**Authors:** 


### Therapeutics.

### PRESCRIPTIONS AND DISPENSING.

Another nitrogenous substance of animal origin, which is sometimes of assistance in making emulsions, is gelatin. This, like casein, and egg-white, contains nitrogen, but, unlike them, it is not a true proteid. A very little gelatin considerably increases the viscosity of aqueous liquids, and this property may be made use of in preparing emulsions. When gelatin is used it is generally in addition to an alkaline salt or some such substance, which must be regarded as the more important agent in emulsifying; the gelatin acts mainly by preserving the emulsion from becoming broken after it has been made. It has not come into very general use in this way, however, although it finds large application in some other branches of dispensing, as we shall see later.

A very usual combination of cod-liver oil is that with malt extract; the latter consists principally of maltose and dextrin, with a smaller proportion of protein. Its action in dividing an oil and keeping the globules apart is no doubt partly due to the protein and to the dextrin, but it is also largely a mechanical effect due to the great viscosity of the extract. If malt extract is stirred in a warmed mortar it becomes considerably more fluid; cod-liver oil is then added gradually, with constant stirring. The quantity of oil may vary very much, even an amount equal to the weight of the extract employed being capable of incorporation. A little gum acacia, or saccharated solution of lime, is sometimes added.

The use of lime water, or other alkaline substances, in making emulsions depends on a chemical change which occurs between the alkali on the one hand and the oil or resin on the other. Fixed oils consist of combinations of one or more fatty acids with glycerol, or some other of the higher alcoholic radicles; by treatment with alkali these combinations are broken up, the alkali combining with the acids, and the glycerin or other alcohol being set free. The compound of the alkali with a fatty acid is a soap, and it is to the soap thus produced that the formation of the emulsion is really due. Most ordinary oils contain more or less of free fatty acids, which combine with the alkali to form soaps before any of the glycerol combination is split up, and in any case the amount of the latter that is actually decomposed is very small indeed. The result is very similar in the case of resins. Instead of thus forming a soap from alkali and oil or resin, a ready-made soap is sometimes employed as an emulsifier, with identical result.

A good illustration of an emulsion of oil made by means of an alkali is given by the castor-oil mixture which was official in the Addendum to the 1885 Pharmacopœia. The formula and directions were:—

| | | |
|---|---|---|
| ℞ Olei ricini | ... | ʒvj. |
| Olei limonis | ... | ♏x. |
| Olei caryophylli | ... | ♏ij. |
| Syrupi | ... | ʒiss. |
| Liquoris potassæ | ... | ʒj. |
| Aquam aurantii floris | ad | ℥ij. |

Mix the oils in a mortar; then incorporate one-third of the solution of potash, and afterwards the syrup; now pour in an additional third of the solution of potash, then, gradually, half of the orange-flower water, then the remainder of the solution of potash, and last of all sufficient orange-flower water to porduce the required volume.

This proportion of alkali, and this method, may also be employed for other oils and oily substances, with slight modifications in different cases. The above formula was abandoned because of its unsuitability as a means of administering castor oil, not because of any faults in the emulsion. The flavouring agents selected and the soapy nature of the product made the mixture very disagreeable; for such an oil as castor oil, which can be perfectly emulsified with the aid of gum, its partial conversion into a soap is not justified.

The official liniments of ammonia and of lime illustrate the production of soaps from alkali and oil, the directions in both cases being merely "shake together." The liniments must be regarded as emulsions, a small part only of the oil being saponified, and the remainder emulsified by means of the soap so produced. The amount of alkali present in either case is not nearly enough to saponify the whole of the oil. Many toilet preparations, such as "lime juice and glycerin," and so forth, belong to the same class. The alkali used in these cases is often borax.

One of the commonest examples of an emulsion produced by the use of alkali with a resinous substance is seen in the way copaiba is usually dispensed. Copaiba, though often called a balsam, is really an oleo-resin—that is, a combination of a resin and an essential oil. On shaking copaiba with diluted solution of potash in a bottle a milky result is obtained at once. The sides of the bottle should be well wetted with the potash before the copaiba is put in. The miscible or soluble Liquor Copaibæ Concentratus is usually made by boiling copaiba with alkali for some time; by this means not only is the resin fully combined with the alkali, but the volatile oil is also driven off. This is detrimental, perhaps, to the therapeutic usefulness of the drug, a point to be borne in mind in prescribing this form of copaiba; but it makes the resulting liquid mix with water without milkiness.

Copaiba may be most elegantly dispensed as an emulsion by the use of both potash and mucilage, as in the following:—

| | | |
|---|---|---|
| ℞ Capaibæ | ... | ʒiv. |
| Liquoris potassæ | ... | ʒij. |
| Mucilaginis acaciæ | ... | ʒij. |
| Spiritus etheris nitrosi | ... | ʒij. |
| Syrupi | ... | ℥ss. |
| Aquam | ad | ℥vj. |

The mucilage should be put into the bottle, and the copaiba added to it by degrees, shaking after each addition; the potash is diluted with an equal volume of water, added to the copaiba, and shaken well; more water is added in successive portions, the spirit and syrup being put in last, diluted together with about an ounce of water.

*(To be continued.)*

